# Dyslipidemia in Peritoneal Dialysis: Implications for Peritoneal Membrane Function and Patient Outcomes

**DOI:** 10.3390/biomedicines12102377

**Published:** 2024-10-17

**Authors:** Natalia Stepanova

**Affiliations:** 1 State Institution “O.O. Shalimov National Scientific Center of Surgery and Transplantology, National Academy of Medical Science of Ukraine”, 03126 Kyiv, Ukraine; n.stepanova@nephrocenter.com; 2 Medical Center “Nephrocenter”, 03057 Kyiv, Ukraine

**Keywords:** dyslipidemia, peritoneal dialysis, peritoneal membrane, outcomes, treatment

## Abstract

Dyslipidemia is a common metabolic complication in patients undergoing peritoneal dialysis (PD) and has traditionally been viewed primarily in terms of cardiovascular risk. Current guidelines do not recommend initiating lipid-lowering therapy in dialysis patients due to insufficient evidence of its benefits on cardiovascular mortality. However, the impact of dyslipidemia in PD patients may extend beyond cardiovascular concerns, influencing PD-related outcomes such as the peritoneal ultrafiltration rate, residual kidney function, PD technique survival, and overall mortality. This review challenges the traditional perspective by discussing dyslipidemia’s potential role in PD-related complications, which may account for the observed link between dyslipidemia and increased all-cause mortality in PD patients. It explores the pathophysiology of dyslipidemia in PD, the molecular mechanisms linking dyslipidemia to peritoneal membrane dysfunction, and summarizes clinical evidence supporting this hypothesis. In addition, this paper examines the potential for therapeutic strategies to manage dyslipidemia to improve peritoneal membrane function and patient outcomes. The review calls for future research to investigate dyslipidemia as a potential contributor to peritoneal membrane dysfunction and to develop targeted interventions for PD patients.

## 1. Introduction

Peritoneal dialysis (PD) is a widely utilized kidney replacement therapy (KRT) for patients with end-stage kidney disease (ESKD) that offers a practical and effective alternative to hemodialysis (HD) [1]. Using the peritoneal membrane as a natural, semi-permeable barrier, PD provides the continuous removal of solutes and fluid, allowing patients to manage ESKD at home [2]. This home-based modality not only provides greater flexibility and a higher quality of life compared to in-center HD, but also allows for the better preservation of residual kidney function (RKF) in many patients [3,4]. However, despite numerous advantages, PD is often associated with metabolic complications, one of which is dyslipidemia [1,3].

The prevalence of dyslipidemia in this population is considerably high and varies between 18.1% and 91.7% across diverse studies [5,6,7]. Moreover, the dyslipidemia observed in PD patients tends to be more atherogenic than that seen in non-dialysis CKD and HD populations [8,9]. This difference is largely attributable to the unique metabolic challenges posed by PD, such as the continuous absorption of glucose from dialysis solution and peritoneal protein losses [10,11]. Furthermore, the use of conventional glucose-based PD solutions can lead to the accumulation of glucose degradation products (GDPs) and the formation of advanced glycation end-products (AGEs), which not only contribute to the atherogenic lipid profile, but negatively affect the peritoneal membrane structure and function [12,13].

The adequate functioning of the peritoneal membrane is essential for the success and longevity of PD as a KRT [2,14]. Over time, however, the integrity of the peritoneal membrane deteriorates, leading to ultrafiltration failure (UFF), peritoneal fibrosis, and ultimately, PD discontinuation [14,15]. The peritoneal membrane’s deterioration, or “aging”, is a complex and multifactorial process [15,16]. One of the primary drivers of membrane damage in long-term PD is chronic exposure to glucose-based dialysis solutions [15,16,17]. The accumulation of GDPs and AGEs causes cellular damage and vasculopathy of the peritoneal microvessels, and triggers inflammation in the peritoneal membrane [15,16]. Alongside the bioincompatibility of PD solutions, uremia per se, and recurrent PD-related infections contribute to the scarring and thickening of the peritoneal membrane, progressively compromising its functionality [14,17]. While these factors are well recognized, the potential contribution of dyslipidemia to peritoneal membrane dysfunction has received less attention.

Traditionally, research on dyslipidemia in PD populations has mainly focused on its impact on cardiovascular morbidity and mortality [5,18,19,20]. This focus is well-founded, as cardiovascular diseases (CVD) remain the leading cause of death in patients undergoing PD [3,21,22]. However, recent studies suggest that dyslipidemia may have broader implications for patient outcomes. Dyslipidemia has been associated with various PD-related complications, including the decline in RKF [23], reduced peritoneal ultrafiltration rate [24], peritonitis treatment failure [25], technique failures [7], and malnutrition [26,27].

Current guidelines do not advocate for initiating lipid-lowering therapy in dialysis patients due to a lack of evidence showing its benefit in reducing cardiovascular mortality [28]. Despite this, evidence supporting the beneficial effects of statins in PD suggests that dyslipidemia may significantly impact these outcomes. Statins have been shown to reduce mortality when initiated before or after starting PD [29,30], inhibit the mesothelial-to-mesenchymal transition (MMT) [31], and mitigate the high-glucose-induced sclerosis of the peritoneal membrane [32]. Their pleiotropic effects also include reducing intraperitoneal inflammation, lowering the incidence of PD-associated peritonitis, and improving dialysis adequacy [33]. All of the findings above indirectly suggest a possible role of dyslipidemia in peritoneal membrane dysfunction. However, the potential effects of dyslipidemia on the peritoneal membrane itself have never been previously explored.

Therefore, this review aims to summarize the current knowledge on the pathophysiology of dyslipidemia in PD, elucidate the molecular mechanisms linking dyslipidemia to peritoneal membrane dysfunction, and discuss the clinical implications of these interactions. It also evaluates current data on potential therapeutic strategies for managing dyslipidemia in patients undergoing PD, with a focus on enhancing peritoneal membrane integrity and function, as well as improving overall patient outcomes.

## 2. Defining Dyslipidemia in PD: Is a One-Size-Fits-All Approach Feasible?

Dyslipidemia represents one of the most prevalent and managed chronic conditions, characterized by abnormal serum levels of cholesterol, triglycerides, or both, often involving disturbances in various lipoprotein types [34,35]. In the general population, it relies on specific predefined values for total cholesterol, triglycerides, low-density lipoprotein (LDL), and high-density lipoprotein (HDL) [36]. However, the applicability of these standard cutoffs to patients undergoing PD is questioned due to their unique metabolic and physiological characteristics. In contrast to the general population, the definition of dyslipidemia in patients undergoing PD remains ambiguous and lacks standardized criteria, presenting significant challenges for both clinical management and research.

Typically, patients undergoing PD have elevated levels of triglycerides, apolipoprotein B (apoB)-containing lipoproteins such as very-low-density lipoproteins (VLDL) and intermediate-density lipoproteins, total cholesterol, and LDL, including small dense LDL and lipoprotein(a) [Lp(a)] [9,37]. Conversely, HDL levels are typically low [9,37]. However, the lipid abnormalities in PD patients extend beyond these traditional markers, as they often experience increases in total free fatty acids, diacylglycerides, triacylglycerides, phosphatidylcholines, phosphatidylethanolamines, ceramides, sphingomyelins, and cholesterol esters [21,38].

A major challenge lies in the absence of standardized thresholds or criteria specifically tailored for the PD population. For example, the 2003 National Kidney Foundation Kidney Disease Outcomes Quality Initiative (KDOQI) guidelines defined atherogenic dyslipidemia based on high LDL levels (≥2.59 mmol/L) and elevated triglycerides (≥2.26 mmol/L) [39]. The 2012 Kidney Disease: Improving Global Outcomes (KDIGO) guidelines, although recommending routine lipid profile measurements in patients with chronic kidney disease (CKD), did not specify a precise definition of dyslipidemia for this population [28]. Cardiology guidelines worldwide have established varying LDL targets for patients with CKD based on their disease stage and cardiovascular risk, with recommendations ranging from <1.4 mmol/L for very-high-risk patients (CKD stages 4–5) to <1.8 mmol/L for high-risk patients (CKD stages 3a–3b) [40,41]. These discrepancies highlight the complexity of applying a uniform definition of dyslipidemia to the CKD cohort in general and to the PD population in particular.

Consequently, determining optimal lipid levels for PD patients remains an area of ongoing debate, with variations often depending on patient characteristics and study findings [42]. For example, a recent retrospective cohort study by Wu et al. identified a U-shaped relationship between the total cholesterol levels and mortality risk in PD patients, suggesting that the lowest mortality risk occurs with total cholesterol levels between 4.1 and 4.5 mmol/L [43]. Similarly, Lin et al. reported a U-shaped association between total cholesterol and mortality, with an increased mortality risk at both very low (<3.89 mmol/L) and very high (>6.48 mmol/L) cholesterol levels, suggesting that this pattern was significant only in patients without RKF [44]. In contrast, those with RKF exhibited a mortality risk pattern more closely aligned with that of the general population, where increasing cholesterol levels correlated with a higher mortality risk [44]. Conversely, Park et al. found that only low total cholesterol levels (<3.89 mmol/L) were associated with increased all-cause mortality [45]. Similar inconsistencies exist regarding the optimal HDL, LDL, and triglyceride levels for PD patients [6,46]. Despite these inconsistencies, numerous studies demonstrate that deviations from normal lipid profiles, grouped under the term dyslipidemia, are strongly associated with adverse clinical outcomes in the PD population, highlighting the need for a fresh perspective on this longstanding issue [5,19,22,43,44,45].

## 3. The Joint Role of CKD and Dialysis in the Development of Dyslipidemia in PD

### 3.1. CKD: A Major Contributor to Dyslipidemia

Dyslipidemia is a common finding in patients with CKD that can develop in the early stages of the disease and becomes particularly pronounced in patients with kidney failure [9,47]. Data from the National Health and Nutrition Examination Survey (NHANES) between 2001 and 2010 highlighted this progression, with dyslipidemia prevalence increasing from 45.5% in CKD stage 1 to 67.8% in stage 4 [48]. Although the mechanisms responsible for CKD-associated dyslipidemia are still not fully understood, the interplay of factors such as uremia-induced oxidative stress, inflammation, and insulin resistance is believed to be the primary drivers of these changes, often manifesting before the initiation of PD [9,49,50].

#### 3.1.1. Key Mechanisms of Dyslipidemia in CKD

Oxidative stress, resulting from an imbalance between the reactive oxygen species (ROS) and antioxidant deficiency, is a central and multifaceted mechanism involved in the development of atherogenic dyslipidemia and its complications [50,51]. A prime example is the oxidative modification of lipoproteins that impairs the functionality and stability of lipoproteins and thus plays a crucial role in the manifestation of dyslipidemia [51,52]. Simultaneously, lipid peroxidation represents another pathway through which oxidative stress disrupts lipid homeostasis, perpetuates the dyslipidemic milieu, and contributes to the complexity of the lipid alterations observed in CKD [51,53]. Nevertheless, the effects of oxidative stress on the lipid profile in CKD are far-reaching and extend to inflammation and insulin resistance. The complex interaction of these factors contributes synergistically to the development of dyslipidemia in CKD [51,52].

The molecular interplay between inflammation and dyslipidemia involves the dysregulation of crucial lipid metabolism enzymes, disrupting lipoprotein homeostasis [9,53,54]. This disruption not only results in elevated triglycerides and reduced HDL, but also fosters heightened levels of atherogenic lipoproteins, collectively increasing the dyslipidemic risk in CKD patients [53]. Inflammation has been demonstrated to modify the association between HDL levels and the risk of major adverse cardiovascular events, diminishing the advantageous link between high HDL levels under an inflammatory influence [55]. Moreover, inflammation is correlated with increased triglycerides and LDL levels, partially attributed to the impact of various cytokines that can suppress the synthesis of lipoprotein lipase, a key enzyme in the triglyceride-rich lipoprotein (TRL) metabolism [56]. Additionally, inflammation can increase the levels of angiopoietin-like protein 4, an inhibitor of lipoprotein lipase activity, further hindering the metabolism of TRL [57]. Dyslipidemia can also contribute to inflammation through various mechanisms, establishing a bidirectional relationship between dyslipidemia and inflammation in CKD [9,50]. Cholesterol, fatty acids, and modified lipids can directly activate inflammatory pathways, triggering inflammatory responses [50,58]. Conversely, proinflammatory signals influenced by cytokines such as interleukin (IL)-1, IL-6, and tumor necrosis factor-alpha (TNF-alpha) have direct effects on the lipid metabolism and contribute to atherogenesis [9,50].

Insulin resistance is another major player in CKD, acting as a bridge between kidney dysfunction and dyslipidemia [59,60]. It promotes dyslipidemia through various mechanisms, including the decreased activity of key enzymes such as lipoprotein lipase (LPL). Decreased LPL activity results in the impaired clearance of TRL, contributing to hypertriglyceridemia [59,60]. In addition, insulin resistance increases the hepatic production of VLDL, further exacerbating hypertriglyceridemia [61]. It also affects the composition and functionality of HDL particles, impairing their atheroprotective properties [60]. This cascade effect leads to the development of atherosclerosis and endothelial dysfunction, further amplifying the risk of CVD in patients with CKD [8,60]. Therefore, the interplay of oxidative stress, inflammation, and insulin resistance creates a complex metabolic environment that profoundly affects the lipid metabolism. These systemic alterations manifest in specific lipid abnormalities that characterize the dyslipidemic profile in patients with CKD. The following key factors contribute to the distinct lipid abnormalities observed in CKD, each stemming from the broader metabolic disturbances previously discussed.

#### 3.1.2. Lipoprotein Modifications in CKD-Induced Dyslipidemia

The diminished clearance of lipoproteins. As mentioned above, in CKD, the activity of LPL, an enzyme that is crucial for the breakdown of TRL such as chylomicrons and VLDL, is often impaired [8,9,47]. This reduced LPL activity leads to the decreased clearance of these lipoproteins from the bloodstream, resulting in elevated triglyceride levels [8,9,62].

Alterations in the structure and function of lipoproteins. CKD induces changes in the size, composition, and function of lipoproteins. For instance, HDL particles become smaller and dysfunctional, losing their anti-inflammatory and antioxidant properties [63,64]. Additionally, the size and mobility of HDL subpopulations are correlated with the estimated glomerular filtration rate [65]. LDL particles also become smaller and denser, which increases their atherogenic potential [64,66]. These structural changes in lipoproteins can impair their ability to interact with cellular receptors, leading to disruptions in lipid homeostasis [9,66].

The modifications and toxicity of lipoproteins. CKD-induced oxidative stress and uremic conditions result in the modification of lipoproteins, producing toxic lipid species that exacerbate the dyslipidemic state [9,64]. For instance, the presence of oxidized LDL (oxLDL) contributes to inflammation and endothelial dysfunction, which further complicates the lipid profile and increases the cardiovascular risk [54]. Additionally, advanced glycation end-products (AGEs), malondialdehyde, and carbamylation contribute to lipoprotein modification, further enhancing their atherogenicity [9,54].

Heightened hepatic activity. Patients with CKD often exhibit hypertriglyceridemia, resulting from both delayed catabolism and the increased hepatic production of VLDL, chylomicrons, and their remnants [9,66]. Additionally, CKD is associated with enhanced hepatic lipogenesis. Elevated fatty acid synthesis in the liver, along with increased levels of key lipogenic enzymes such as fatty acid synthase and acetyl-CoA carboxylase, contributes to the accumulation of lipids in both the liver and circulation [67].

Together, CKD serves as a significant prelude to dyslipidemia, with its impact becoming increasingly pronounced with kidney function decline. The interplay of uremia-induced oxidative stress, inflammation, and insulin resistance forms the foundation of specific lipid abnormalities, often manifesting before the initiation of PD.

### 3.2. PD: A New Layer in Dyslipidemia

PD introduces additional metabolic complexities that can further exacerbate dyslipidemia in patients with CKD. A recent study by Kane et al. demonstrates the pronounced effect of PD on atherosclerosis progression in uremic conditions, where PD exposure was found to accelerate atherosclerosis by triggering systemic inflammation [68]. PD initiation in uremic mice led to the development of larger, more advanced, and vulnerable plaques, as well as faster new plaque formation. While CKD alone showed a tendency toward increased plaque burden and inflammation, the combination of CKD and PD significantly amplified these effects compared to the controls [68]. A critical factor in this process is the continuous exposure of the peritoneal membrane to glucose-based PD solutions, along with the loss of proteins in the dialysate, which further influences lipid profiles in PD patients [9,21,47].

One of the most significant contributors to dyslipidemia in PD patients is the continuous absorption of glucose from the dialysis solution. Studies have shown that daily glucose absorption can vary from 89 to 316 g per day, depending on the type of PD (continuous ambulatory or automated PD), dwell time, and the glucose concentration in the solution [69,70]. Glucose absorption may constitute a considerable proportion of a patient’s daily caloric consumption, thereby contributing to hyperglycemia and insulin resistance [69,70]. Subsequently, insulin resistance stimulates hepatic lipogenesis and enhances the production of VLDL cholesterol. This process is associated with elevated levels of triglycerides and cholesterol, particularly VLDL and LDL [71,72]. It has been recently demonstrated that increased peritoneal glucose absorption correlates with elevated cholesterol levels, with a 10 g/d increase linked to a 0.145 mmol/L rise in cholesterol [73]. Conversely, the use of low-glucose PD solutions has significantly improved serum triglyceride, VLDL, and ApoB lipoprotein levels compared with high-glucose PD solutions [74]. It should be emphasized that although peritoneal glucose absorption has been considered a major contributor to dyslipidemia in PD, some research indicates no significant change in patients’ serum lipid profile even with a median glucose absorption of 172.5 (75.5–265.5) mmol/day [10]. These mixed findings suggest that the relationship between glucose absorption and dyslipidemia may be complex and influenced by additional factors. Furthermore, the persistence of hyperglycemia can also promote the non-enzymatic glycation of proteins, resulting in the formation of AGEs [75]. Elevated levels of AGEs disrupt normal cellular functions and promote inflammation, oxidative stress, and vascular smooth muscle apoptosis, all of which are key factors in atherosclerosis [76].

In addition to glucose absorption, protein loss through the peritoneal membrane is thought to be the second most important factor contributing to the development of dyslipidemia in PD. During PD, significant amounts of protein (4–10 g per day) are lost in the dialysate, which can lead to hypoalbuminemia and prompt the liver to increase the production of lipoproteins as a compensatory mechanism [77,78]. This hepatic response exacerbates the dyslipidemic profile by increasing the levels of LDL and VLDL. In addition, Eibensteiner et al. have identified 549 proteins lost via transperitoneal diffusion, including those involved in HDL formation, indicating a direct link between peritoneal protein loss and HDL metabolism [79]. Lastly, protein loss in PD patients has been suggested to exacerbate malnutrition and inflammation, both of which are linked to dyslipidemia [80,81]. However, it is important to note that recent studies have questioned the direct association between daily protein loss, malnutrition, and inflammation, suggesting that these relationships may be more complex than previously understood [77].

The impact of PD on dyslipidemia is further complicated by other interconnected factors that contribute to increased oxidative stress, inflammation, and insulin resistance. For example, an erythropoietin deficiency, which accelerates oxidative stress and the chronic inflammatory response, has been shown to alter serum lipid profiles, whereas erythropoietin administration has been found to reduce total cholesterol, triglyceride, and LDL levels [82,83]. Similarly, a vitamin D deficiency is associated with an increased risk of dyslipidemia, and its supplementation has been shown to have beneficial effects on lipid profiles [84,85]. Additionally, the decline in RKF also is a significant factor in the progression of dyslipidemia in patients undergoing PD. As RKF decreases, there is a concomitant increase in inflammation and oxidative stress, which can have detrimental effects on lipid profiles [86,87]. The loss of RKF is associated with the accumulation of uremic toxins, which can interfere with the lipid metabolism and contribute to the development of dyslipidemia [23,44]. Furthermore, PD-related peritonitis can significantly influence the lipid metabolism, and the relationship is bidirectional. Ye et al. have demonstrated that peritonitis episodes affect the association between LDL and cardiovascular outcomes [88]. Conversely, the presence of diabetes coupled with low HDL levels, and a high triglyceridis or C-reactive protein/HDL ratio have been found to predict the risk of PD-related peritonitis [89,90,91]. It should be noted that peritonitis episodes, as well as PD treatment itself, can further alter the gut microbiota, contributing to the complex dyslipidemic profile observed in these patients [92,93]. The peritoneal environment and the absorption of glucose-based PD solutions can disrupt the gut barrier and exacerbate dysbiosis, leading to the acceleration of pre-existing chronic inflammation and insulin resistance [94].

**Figure 1:**
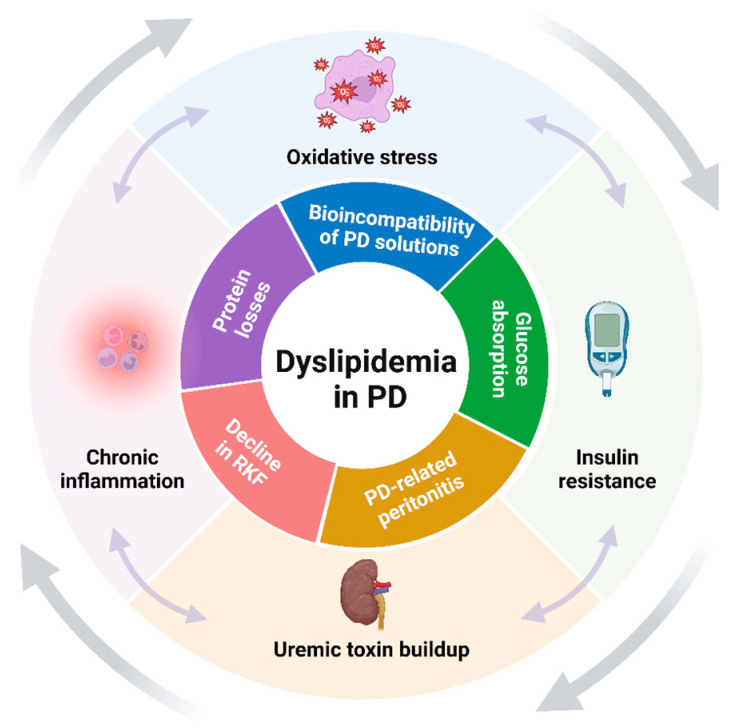
Key synergistic factors contributing to dyslipidemia in PD (created with BioRender.com). The figure highlights the primary factors that drive the development and progression of dyslipidemia in patients undergoing PD. The factors are schematically categorized into those related to CKD (outer layer) and those specific to PD (inner layer), illustrating their combined impact on lipid metabolism. Abbreviations: PD, peritoneal dialysis; RKF, residual kidney function.

Thus, the combined effect of CKD and PD-specific factors creates a unique dyslipidemic environment that is more atherogenic and challenging compared to that seen in patients with non-dialysis CKD or those treated with HD (Figure 1). As such, dyslipidemia in patients undergoing PD is not merely a secondary condition but may be a significant contributor to their clinical outcomes.

## 4. Introduction to Structural and Functional Dynamics of the Peritoneal Membrane in PD

The aging of the peritoneal membrane in PD has been widely studied and documented, highlighting the structural and functional alterations that occur over time [2,14,15,95].

The peritoneal membrane has a complex, multilayered structure comprising a mesothelial cell layer, a submesothelial compact zone, and a vascular layer [2,14,96]. The mesothelial cell layer forms the superficial part of the peritoneum and serves as a crucial barrier that regulates fluid and solute exchange while protecting the underlying tissues from infection and inflammation. Beneath the mesothelial layer is the submesothelial compact zone, which is rich in extracellular matrix (ECM) components such as fibroblasts, mast cells, and collagen, providing essential structural support and contributing to tissue rigidity and resilience. The deepest layer, the vascular layer, contains a network of capillaries embedded in fatty connective tissue, which supplies the peritoneum with blood flow estimated at 60–100 mL/min [97].

In PD, the primary role of the peritoneal membrane is to facilitate the exchange of metabolites, uremic toxins, and fluid between the blood in the peritoneal capillaries and the dialysis solution in the peritoneal cavity through diffusion, osmosis, and convection [2,15]. However, with prolonged use of PD, the peritoneal membrane undergoes progressive structural and functional changes [2,15]. In response to uremia per se, PD solution incompatibility, bacterial infection, or mechanical stress, the number of mesothelial cells decreases or they may be completely lost [2,14,15,98].

The loss of mesothelial cells is closely associated with the disruption of apical–basal polarity and cell adhesion, which triggers the MMT [2,14,15]. During the MMT, mesothelial cells lose their epithelial characteristics, including intercellular junctions, as evidenced by the reduced expression of adhesion proteins like E-cadherin and zona occludens 1 (ZO-1) [14,15]. Concurrently, there is an increased expression of mesenchymal markers such as N-cadherin, vimentin, and α-smooth muscle actin (α-SMA), leading to the acquisition of a myofibroblast-like phenotype [95]. This transition significantly contributes to the thickening of the submesothelial compact zone, from its physiological thickness, ultimately resulting in peritoneal fibrosis [96,99].

The progression of these structural changes is driven by various molecular mechanisms, including pseudohypoxia and the activation of the renin–angiotensin–aldosterone system (RAAS), which further exacerbate tissue remodeling [15,96,99]. Additionally, the release of inflammatory mediators such as tumor necrosis factor α (TNF-α), IL-1β, IL-6, IL-8, IL-17, monocyte chemotactic protein (MCP)-1, adhesion molecules, and vascular endothelial growth factor (VEGF) contributes to a chronic inflammatory state that perpetuates membrane damage [14,87,96]. This inflammatory cascade activates a complex network of cellular signaling pathways, including transforming growth factor (TGF) β/Smad and non-Smad signaling, nuclear factor-kappa B (NF-κB), Wnt, and Notch pathways, which further promote the MMT and fibrosis [96,99,100].

Moreover, the vascular layer undergoes significant alterations during the aging process, such as neovascularization and progressive subendothelial hyalinization, leading to luminal narrowing or complete obliteration—a condition collectively termed vasculopathy [14,15]. These vascular changes are directly associated with clinical manifestations of peritoneal dysfunction, including increased peritoneal transport rates and reduced ultrafiltration [101].

## 5. Gut Microbiota as a Key Contributor to Dyslipidemia and Peritoneal Membrane Dysfunction in PD

The gut microbiota plays a crucial role in modulating dyslipidemia [102,103] and is believed to influence peritoneal membrane integrity in patients undergoing PD [94,104]. Dysbiosis, characterized by reduced microbial diversity and an imbalance between beneficial and harmful bacteria, is common in these patients [105]. Key factors contributing to gut microbiota dysbiosis include the uremic milieu, comorbidities, dietary restrictions (particularly the limited intake of fiber-rich foods), medication use (such as antibiotics and phosphate binders), glucose absorption from PD solutions, increased intra-abdominal pressure, and the presence of a PD catheter itself [106,107,108].

Overall, gut microbiota alterations in PD patients can be characterized by a decrease in beneficial bacteria such as *Bifidobacterium* and *Lactobacillus* species, which are known for their anti-inflammatory properties and ability to produce short-chain fatty acids (SCFAs) [92,105]. There is also a reduction in other SCFA-producing bacteria, including members of the *Ruminococcaceae* and *Lachnospiraceae* families [105]. Conversely, an over-representation of pathogenic and pro-inflammatory bacteria such as the *Escherichia coli* and *Enterococcus* species is frequently observed, along with an increase in urease-producing bacteria like *Proteus* and *Klebsiella* [105].

The gut microbiota influences the lipid metabolism by metabolizing, transforming, and hydrolyzing complex lipids, thereby modulating host lipid homeostasis [102,109]. A key mechanism involves the interaction with bile acids. Bile acids, synthesized from cholesterol in the liver, are essential for the digestion and absorption of dietary fats [103]. Gut bacteria modify primary bile acids into secondary bile acids, which act on receptors like the farnesoid X receptor and G protein-coupled bile acid receptor to regulate the lipid metabolism. Dysbiosis can alter bile acid profiles, disrupting these regulatory pathways and leading to impaired lipid absorption and increased circulating lipid levels, contributing to dyslipidemia [103,110]. Another key pathway involves SCFAs such as acetate, propionate, and butyrate, which are produced by the fermentation of dietary fibers by gut bacteria [109,110]. SCFAs play a crucial role in the lipid metabolism by modulating the expression of genes related to lipid synthesis and breakdown in the liver. Changes in SCFA production due to dysbiosis can disrupt lipid homeostasis, promoting the development of dyslipidemia [103,110]. Moreover, microbial metabolites such as LPS and trimethylamine nitrogen oxide (TMAO) can impact lipid metabolism pathways and exacerbate chronic inflammation, contributing to atherogenic dyslipidemia and CVD [103].

Importantly, although many studies have identified associations between gut microbiota and lipid profile markers in the general population [111,112], the specific role of gut microbiota in the development of dyslipidemia in PD patients remains unclear. Similar to findings in the general cohort, Guo et al. observed that microbial diversity in PD patients was negatively correlated with triglyceride levels and positively correlated with HDL levels [93]. Wang et al. also reported that lipid profile markers were associated with the abundance of specific gut bacteria, such as *Ruminococcus2*, *Dorea*, and *Olsenella* [113]. Moreover, our recent report has shown a strong association between gut microbiota alterations, dyslipidemia, increased serum indoxyl sulfate levels, heightened oxidative stress, and elevated serum IL-6 and MCP-1 levels, which together contribute to a high three-year CVD risk in patients with ESKD [114]. In contrast, Hobson et al. found that multiple uremic toxins were negatively associated with lipid biomarkers in patients with ESKD, even after adjusting for clinically relevant covariates [49]. Additionally, Li et al. reported a direct correlation between triglycerides and fecal SCFA levels alongside an inverse correlation between TMAO and LDL levels [105]. These findings highlight the complex and sometimes conflicting relationships between gut-derived metabolites and lipid profiles in PD patients.

The gut microbiota has also been suggested to play a role in peritoneal membrane integrity and function in PD patients [104]. Low gut microbiota diversity has been independently associated with a higher risk of PD technique failure [93], and elevated circulatory levels of LPS have been negatively associated with peritoneal Kt/V [105]. Additionally, p-cresyl sulfate has been identified as an independent predictor of PD technique failure over a 5-year follow-up period [115]. Despite these clinical observations, the precise mechanisms underlying the relationship between the gut microbiota and peritoneal membrane integrity remain unclear.

One hypothesis is that increased gut permeability allows the translocation of bacteria and their byproducts into the bloodstream and peritoneal cavity, potentially causing the inflammation and fibrosis of the peritoneal membrane. Supporting this idea, similarities between the gut and peritoneal microbiomes have been observed in PD patients [104]. Moreover, severe intestinal epithelial dysfunction has been reported following long-term exposure to PD solutions in both control and 5/6Nx animal models of PD [116]. On the other hand, a recent study by Li et al. found no correlation between gut permeability markers, circulating bacterial fragments, and hyperhydration in PD patients [117]. Nonetheless, both circulating bacterial fragments and gut permeability markers were strongly associated with insulin resistance parameters.

Another hypothesis involves the systemic impact of uremic toxins on the peritoneal membrane. Elevated levels of indoxyl sulfate and p-cresyl sulfate, associated with dysbiosis, contribute to chronic inflammation, endothelial dysfunction, and fibrosis [118], thereby contributing to structural alterations in the peritoneal membrane. TMAO has been shown to directly induce peritoneal mesothelial cell necrosis, together with an increased production of pro-inflammatory cytokines including TNF-α, IL-6, and IL-1β [119]. In addition, reduced levels of SCFAs may weaken the peritoneal barrier, making it more vulnerable to injury from inflammatory mediators and mechanical stress during dialysis. For instance, Jiang et al. demonstrated that alterations in the gut microbiota and decreased SCFA production were associated with prolonged dialysis duration, high peritoneal glucose exposure, and a loss of RKF in PD patients [92].

Overall, these findings underscore the multifaceted role of the gut microbiota in modulating both the lipid metabolism and peritoneal membrane integrity in PD patients. However, the complex and sometimes contradictory nature of these interactions highlights the need for further research.

## 6. Possible Mechanisms of Dyslipidemia-Mediated Peritoneal Membrane Damage

The impact of circulating lipoproteins on the structure and function of the peritoneal membrane is an area that has not been extensively studied, yet emerging evidence suggests that dyslipidemia may play a critical role in peritoneal membrane damage.

One of the earliest insights into the local impact of dyslipidemia on the peritoneal membrane came from a study by Henkin et al., which revealed significantly higher concentrations of VLDL and apoB in the PD effluent (PDE) in dyslipidemic patients compared to normolipidemic ones [120]. Later, Fang et al. showed that human peritoneal mesothelial cells (HPMCs) can uptake LDL via LDL receptors (LDLR) on their cell membrane [121]. Moreover, the authors demonstrated that exposure to LDL in high-glucose conditions induced morphological changes in HPMCs, causing the MMT, evidenced by the increased expression of α-SMA and decreased E-cadherin expression. In addition, LDL exposure led to the increased production of extracellular matrix components, particularly type I collagen (Col I), and the upregulation of plasminogen activator inhibitor-1 (PAI-1), which inhibits ECM degradation [121]. The authors suggested that LDL, especially in the presence of high glucose concentrations typical in PD, can promote the MMT in HPMCs and contribute to extracellular matrix accumulation, potentially leading to peritoneal fibrosis. Conversely, HDL and its main protein component, apoA-I, appear to have protective effects against peritoneal fibrosis. Lu et al. have demonstrated that apoA-I levels are positively correlated with better peritoneal ultrafiltration function in patients undergoing PD [122]. However, although HDL cholesterol constitutes 50% to 60% of the total cholesterol in PDE [120], the altered lipid environment may impair HDL’s protective functions, reducing its effectiveness and potentially contributing to membrane damage and dysfunction.

**Figure 2:**
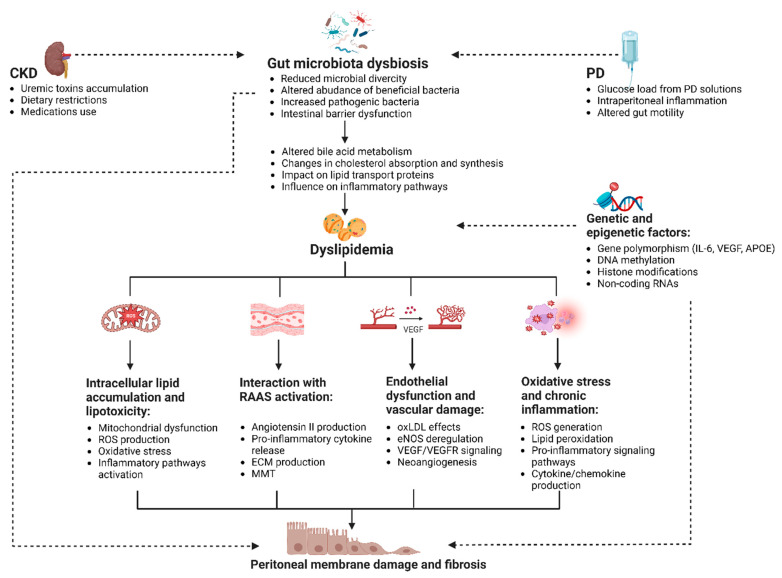
Mechanisms linking dyslipidemia and peritoneal membrane damage in PD (created with BioRender.com). The figure illustrates the interplay between dyslipidemia and peritoneal membrane dysfunction in patients undergoing PD. Dyslipidemia may contribute to peritoneal membrane damage through several pathways, including intracellular lipid accumulation and lipotoxicity, interaction with the RAAS, endothelial dysfunction, oxidative stress, and inflammation. The figure highlights the role of gut dysbiosis and glucose-based PD solutions in exacerbating dyslipidemia and its potential impact on the peritoneal membrane. Key processes in this interaction include mitochondrial dysfunction, pro-inflammatory signaling pathways, ECM production, MMT, and neoangiogenesis. It also underscores the importance of genetic and epigenetic factors in influencing individual susceptibility to these processes. Abbreviations: APOE, apolipoprotein E; CKD, chronic kidney disease; DNA, deoxyribonucleic acid; ECM, extracellular matrix; eNOS, endothelial nitric oxide synthase; IL-6, interleukin 6; MMT, mesothelial-to-mesenchymal transition; oxLDL, oxidized low-density lipoprotein; PD, peritoneal dialysis; RAAS, renin–angiotensin–aldosterone system; RNAs, ribonucleic acids; ROS, reactive oxygen species; VEGF, vascular endothelial growth factor; VEGFR, vascular endothelial growth factor receptor.

Nonetheless, the adverse effects of dyslipidemia on the peritoneal membrane are not limited to the direct accumulation of lipids in the PDE; a complex network of additional pathways should also be considered (Figure 2).

### 6.1. Intracellular Lipid Accumulation and Lipotoxicity

Dyslipidemia may significantly impact the intracellular lipid metabolism within mesothelial cells, leading to a condition known as lipotoxicity [123,124]. Although specific studies on HPMCs are limited, the general concept of lipotoxicity suggests that the excessive intracellular accumulation of lipids such as triglycerides, cholesterol esters, and other lipid derivatives can overwhelm cellular mechanisms and induce dysfunction and apoptosis [123,124]. Lipotoxicity leads to mitochondrial overload, disrupting normal energy production and causing a decline in mitochondrial efficiency. Mitochondrial dysfunction is a well-established factor in peritoneal membrane damage observed in PD [125,126]. A recent study by Ramil-Gómez et al. demonstrated a significant increase in mitochondrial ROS production and a loss of mitochondrial membrane potential in mesothelial cells exhibiting a fibroblast-like phenotype [126]. In addition, the authors quantified mitochondrial DNA levels in the supernatant of PD effluent and found an inverse correlation with peritoneal ultrafiltration.

Increased ROS production due to mitochondrial dysfunction exacerbates oxidative stress, leading to further cellular damage and apoptosis [124]. ROS, being highly reactive, can damage cellular components such as lipids, proteins, and DNA, which in turn triggers various stress responses and inflammatory pathways [127]. This oxidative environment aggravates cellular dysfunction and accelerates peritoneal membrane damage [128].

Moreover, excessive ROS can activate pro-inflammatory signaling pathways, such as NF-κB, and trigger the release of damage-associated molecular patterns (DAMPs) from mitochondria, including mitochondrial DNA [129,130]. DAMPs act as inflammatory mediators, further exacerbating the inflammatory response. Conversely, inflammation can exacerbate oxidative stress by activating immune cells that produce additional ROS, creating a feedback loop between oxidative stress and inflammation [129,130]. Supporting these mechanisms, Xie et al. demonstrated a significant association between mitochondrial DNA levels in PD effluent and peritoneal solute transport rates, along with elevated concentrations of inflammatory markers such as IL-6 and TNF-α, underscoring the interconnected roles of lipotoxicity, mitochondrial dysfunction, and inflammation [125].

The cascading consequences of this pathological cycle culminate in peritoneal membrane fibrosis. Research by Liu et al. has illuminated the relationship between intracellular lipid accumulation and extracellular matrix accumulation resulting in fibrosis [131]. Specifically, they found that the deposition of intracellular cholesterol, triggered by exposure to high-glucose PD solutions, stimulates the increased expression of α-SMA and Col I, ultimately leading to the thickening and scarring of the peritoneal membrane [131]. Nevertheless, despite the growing evidence for intracellular lipid accumulation and lipotoxicity’s role in peritoneal membrane damage, many critical aspects remain unexplored. For example, the specific lipid species driving lipotoxic signaling in HPMCs, the impact of lipid-induced mitochondrial dysfunction, and the potential crosstalk between the lipid metabolism and other cellular stress pathways are still unknown. These gaps in knowledge highlight the need for further research into the specific mechanisms of lipotoxicity in HPMCs.

### 6.2. RAAS Activation

Dyslipidemia and high glucose are known to influence systemic RAAS activation, a critical regulator of blood pressure, fluid balance, and inflammation [132]. Elevated levels of circulating lipids, particularly LDL and triglycerides, have been associated with the increased activity of RAAS components such as angiotensin II (Ang II) [132,133]. Ang II, a potent vasoconstrictor, promotes endothelial dysfunction, oxidative stress, and inflammation, all of which are linked to atherogenic dyslipidemia [132,133]. Although RAAS activation is traditionally associated with systemic effects, it also contributes to a pro-inflammatory and pro-fibrotic milieu within the peritoneal environment [134,135,136]. Ang II promotes the production of pro-inflammatory cytokines such as IL-6 and TGF-β1, and increases oxidative stress, thereby exacerbating inflammation in the peritoneal cavity [136,137]. Ang II can also directly impact mesothelial cells by inducing the MMT and enhancing the expression of profibrotic markers, increasing ECM production [136,137].

RAAS activation interacts synergistically with dyslipidemia, creating a vicious cycle where lipid abnormalities and RAAS activation mutually reinforce each other [133]. This synergy accelerates peritoneal fibrosis, compromises membrane function, and heightens the risk of ultrafiltration failure in PD patients [136]. Additionally, Ang II-mediated oxidative stress and inflammation lead to increased vascular permeability, facilitating the infiltration of inflammatory cells into the peritoneum and amplifying tissue damage [136].

In a recent report series, Liu et al. have highlighted the role of local RAAS activation in the peritoneum under high-glucose conditions [134,135]. Their findings suggest that high-glucose PD solutions stimulate intracellular Ang II/Ang II type 1 receptor (AT1) signaling. This signaling disrupts the LDLR’s negative feedback regulation, leading to lipid deposition in HPMCs and promoting ECM production. Interestingly, the protective effects of AT2 signaling on HPMCs were associated with decreased intracellular lipid deposition, indicating a potential pathway for mitigating RAAS-mediated peritoneal damage [134,135].

### 6.3. Endothelial Dysfunction and Vascular Damage

The primary mechanisms by which dyslipidemia leads to endothelial dysfunction involve oxLDL, which suppresses nitric oxide (NO) production, enhances the expression of leukocyte adhesion molecules, inhibits endothelial cell migration, and induces the apoptosis of endothelial cells [138,139]. The resulting oxidative stress leads to the deregulation of endothelial nitric oxide synthase (eNOS) activity and the inactivation of NO, further compromising the endothelial function [139]. Although specific research on the peritoneal membrane is limited, early studies have shown that in long-term PD patients, there is an upregulation of eNOS activity, which is linked to increased nitrotyrosine formation and a 2.5-fold increase in the vascular density and endothelial area in the peritoneum [140,141]. The studies suggest that dyslipidemia may contribute to peritoneal vascular changes through similar mechanisms seen in systemic vascular dysfunction.

Vascular endothelial growth factor receptor (VEGFR) signaling plays a crucial role in the inflammatory processes involved in vascular dysfunction and atherosclerosis [142]. VEGFR activation promotes endothelial cell permeability and leukocyte adhesion, facilitating inflammatory cell infiltration into the vessel wall. It also induces the expression of adhesion molecules like vascular cell adhesion molecule-1 (VCAM-1) and intercellular adhesion molecule-1 (ICAM-1) on endothelial cells and stimulates the production of pro-inflammatory cytokines and chemokines [139].

In the context of the peritoneal membrane, VEGF and VEGFR are key angiogenic mediators involved in peritoneal neoangiogenesis [143,144]. Altered mesothelial cells, especially under high-glucose conditions and other pathological stresses such as inflammation and hypoxia, are the primary sources of VEGF in the peritoneum [143]. Other cells, including vascular endothelial cells, macrophages, and adipocytes, also contribute to VEGF secretion through various signaling pathways, such as TGF-β, Wnt/β-catenin, and Notch, thereby promoting peritoneal neoangiogenesis [143,144]. For example, Kariya et al. demonstrated that neoangiogenesis is linked to fibrosis via the TGF-β1-VEGF-A pathway in mesothelial cells and fibroblasts [144]. Furthermore, their study showed a direct association between the VEGF-A concentration and the dialysate-to-plasma creatinine ratio (D/P Cr), as well as TGF-β1 levels in human PDE. VEGF-A mRNA levels were significantly elevated in the peritoneal tissues of patients with UFF and correlated with an increased number of vessels and greater peritoneal thickness [144].

### 6.4. Oxidative Stress and Inflammatory Responses

Dyslipidemia may adversely affect peritoneal membrane health by intensifying existing oxidative stress and chronic inflammation. These processes interact complexly, exacerbating tissue damage and impairing membrane function [51]. As outlined above, oxidative stress is a primary mechanism through which dyslipidemia may affect the peritoneal membrane [24,98]. Elevated levels of oxLDL in the peritoneal cavity can trigger a cascade of events that generate ROS and promote the lipid peroxidation of membrane phospholipids [145]. This cascade is supported by extensive data linking dyslipidemia with increased blood levels of oxidative stress markers in patients undergoing PD [24,146]. A recent study by Papadea et al. has indicated increased serum levels of oxLDL-specific markers, such as cholesteryl ester-OOH, triglyceride-OOH, and free cholesterol-OOH in patients undergoing PD compared to controls and HD patients [147]. Notably, most of these markers were inversely correlated with LDL particle numbers. In our recent study, dyslipidemia was found to be significantly associated with increased levels of oxidative stress markers in the blood and a reduction in antioxidant markers [24]. In parallel, UFF was also linked to elevated oxidative stress markers. After adjusting for potential confounders, a significant interaction between dyslipidemia and the peritoneal ultrafiltration rate was observed, suggesting that their combined effect further amplifies oxidative stress [24]. Despite the strong association between dyslipidemia and circulation oxidative stress markers, their direct measurements in PDE are limited by two studies. Latcha et al. have shown that elevated levels of oxidized proteins in PDE correlate with a higher peritoneal transport status and a loss of RKF in PD patients [148]. Similarly, Morinaga et al. have found that elevated PDE free radicals are associated with RKF decline and PD technique failure in PD patients [149].

Concurrently, dyslipidemia may be one of the other significant factors that can exacerbate the inflammatory environment and contribute to peritoneal membrane damage. Although direct evidence linking dyslipidemia, inflammation, and peritoneal damage is also limited, there is substantial research demonstrating the pro-inflammatory effects of dyslipidemia in atherosclerosis [150,151]. Dyslipidemia is known to activate key pro-inflammatory signaling pathways, such as NF-κB, NLRP3 inflammasome, Notch and Wnt, and toll-like receptor (TLR) pathways, particularly TLR2 and TLR4 [150,151]. These pathways are typically activated in response to DAMPs from stressed or damaged cells, AGEs, and oxLDL particles, all of which contribute to peritoneal membrane damage in PD [14,95,99].

In the broader context of chronic diseases, dyslipidemia promotes the recruitment of inflammatory cells, such as macrophages, to affected tissues, where they release pro-inflammatory cytokines and chemokines, including TNF-α, IL-1β, IL-6, IL-8, MCP-1, VEGF, and adhesion molecules [52,150,151]. This cytokine milieu creates a state of chronic low-grade inflammation that can contribute to tissue fibrosis and damage [150,151]. While these mechanisms have been extensively studied in cardiovascular and metabolic diseases, they likely have parallels in the peritoneal membrane environment. It has been demonstrated that PD patients with dyslipidemia had significantly higher levels of IL-10, TNF-α, and MCP-1 in PDE compared to dyslipidemia-free patients [86]. Moreover, PDE TNF-α concentrations were inversely correlated with serum HDL levels, while PDE MCP-1 concentrations were directly associated with the atherogenic index of plasma (AIP) and predicted PD inadequacy [86].

Nevertheless, high-glucose conditions inherent to PD, along with its byproducts (AGEs and GDPs), are the primary drivers of systemic and intraperitoneal oxidative stress, and inflammation that can directly induce peritoneal membrane fibrosis [16,152]. Simultaneously, high-glucose PD solutions are also a well-documented cause of hyperglycemia and insulin resistance, leading to an altered lipid metabolism and the accumulation of circulating lipids [14,16,73,98,153]. This interplay creates a cycle of metabolic disruption, where an altered lipid metabolism exacerbates inflammation, and the resulting inflammatory processes further disrupt the lipid balance, leading to ongoing peritoneal membrane damage.

### 6.5. Genetic and Epigenetic Factors

Genetic and epigenetic factors significantly influence individual susceptibility to both dyslipidemia [154,155] and peritoneal membrane dysfunction [156,157]. These factors influence the expression of genes involved in the lipid metabolism, inflammation, oxidative stress, and fibrosis, thereby contributing to variations in clinical outcomes among PD patients [154,156,157,158].

#### 6.5.1. Genetic Factors

Polymorphisms in genes related to peritoneal membrane function play a significant role in determining individual susceptibility to PD-related complications [156]. Genetic variations can influence the baseline characteristics of the peritoneal membrane [156,159]. One of the most well-established genetic influences on peritoneal membrane function involves polymorphisms in the *IL-6* gene [156,159]. Multiple studies have consistently demonstrated that *IL-6* gene polymorphisms affect the peritoneal membrane solute transport rate [156,159,160]. Angiogenesis-related genes have also been implicated in peritoneal membrane function. Specifically, the CC genotype of *VEGF* rs3025039 and the AA genotype of *KDR* rs2071559 have been associated with a high peritoneal transport status [161]. The HH genotype of the *CYP11B2* gene has been associated with a significantly lower serum IL-6, which correlates with lower concentrations of PAI-1 and VEGF [162].

Other inflammatory genes, such as *IL-1β* and *TGF-β1*, though not as extensively studied as IL-6, may also influence peritoneal membrane health. Polymorphisms in the *IL-1β* gene have been linked to an increased risk of PD-related peritonitis [163]. Conversely, the presence of the C allele in the *TGF-β1* rs1800471 polymorphism might offer a protective effect against the development of encapsulating peritoneal sclerosis [164].

Furthermore, although not directly related to peritoneal function, polymorphisms in genes involved in the lipid metabolism, such as apolipoprotein E (*APOE*), *LDLR*, and proprotein convertase subtilisin/kexin type 9 (*PCSK9*), may indirectly impact the peritoneal membrane by altering systemic lipid profiles. For example, the E4 allele of the *APOE* gene is associated with higher LDL and lower HDL levels [165], which may contribute to lipid accumulation in the peritoneal membrane. Although direct studies on *APOE* and peritoneal fibrosis are lacking, related genetic mechanisms, such as the role of the enhancer of zeste homolog 2 (*EZH2*) in regulating TGF-β signaling pathways, suggest a connection between APOE’s functions in inflammation and the lipid metabolism and its potential influence on fibrosis [166]. Mutations in the *LDLR* gene, which impair the uptake and clearance of LDL, can lead to familial hypercholesterolemia [167] and are also associated with accelerated peritoneal membrane damage and fibrosis [121,135]. Variants in the *PCSK9* gene, known to increase LDL levels, have been linked to an increased risk of subclinical atherosclerosis [155], with elevated plasma PCSK9 levels directly correlated with LDL levels in patients undergoing PD [168].

It is important to note that while these genetic variations are associated with dyslipidemia and related processes, their direct impact on the peritoneal membrane has not been extensively studied. The interplay between these genetic factors and environmental influences ultimately determines an individual’s lipid profile and its potential effects on various tissues, including the peritoneal membrane.

#### 6.5.2. Epigenetic Modifications

The impact of dyslipidemia on the peritoneal membrane may be also mediated through epigenetic mechanisms. Unlike genetic mutations, epigenetic modifications can be reversible and may provide potential targets for therapeutic intervention [157,158]. Epigenetic mechanisms, such as DNA methylation, histone modifications, and non-coding RNA interactions can influence gene expression without altering the underlying DNA sequence [154,158]. DNA methylation, one of the most studied epigenetic processes, has been implicated in the regulation of lipid-related genes. For example, methylation patterns in genes such as *CPT1A, ABCG1*, and *SREBF1* have been associated with altered lipid profiles [154]. A genome-wide study identified novel associations between DNA methylation patterns and circulating lipid levels, providing insights into potential therapeutic targets [169].

Histone modifications also play a role in regulating lipid-related genes. Studies have shown that histone acetylation and deacetylation can influence the expression of genes involved in cholesterol metabolism [154,158]. Additionally, histone methylation patterns have been associated with lipid phenotypes in animal models, further highlighting the importance of these epigenetic marks in lipid regulation [154].

Non-coding RNAs, including microRNAs (miRNAs) and long non-coding RNAs (lncRNAs), have emerged as important regulators of lipid homeostasis. For example, miR-33a/b has been identified as a key regulator of cholesterol efflux and fatty acid oxidation [154,158].

Similarly, in peritoneal membrane fibrosis, epigenetic changes can influence the expression of pro-fibrotic and pro-inflammatory genes, promoting extracellular matrix deposition, the MMT, and vascular remodeling [157]. Epigenetic alterations in key regulators of fibrosis, such as TGF-β, Smad proteins, and other signaling molecules, can exacerbate the fibrotic response, increasing susceptibility to peritoneal membrane dysfunction [157,170].

Furthermore, environmental factors, such as diet, uremic toxins, and exposure to PD solutions, can interact with an individual’s epigenetic landscape, further influencing susceptibility to both dyslipidemia and peritoneal fibrosis [157,158]. The interplay between genetic predispositions and epigenetic modifications creates a complex network: genetic variants influencing the lipid metabolism may interact with environmental factors to induce epigenetic changes, which in turn affect mesothelial cell function and contribute to fibrosis.

## 7. Clinical Evidence of Dyslipidemia Impact on PD-Related Patients’ Outcomes

A growing body of clinical evidence suggests that dyslipidemia can significantly impact PD-related outcomes, such as the peritoneal ultrafiltration rate, RKF, technique survival, and overall patient survival. The association between different lipid markers and the outcomes is detailed in Table 1.

As summarized in Table 1, elevated triglyceride levels have been associated with reduced technique survival and a higher risk of PD-related peritonitis [105]. It also correlated with the treatment failure of PD-related peritonitis and the overall survival rate, highlighting the importance of maintaining optimal lipid levels to enhance the longevity and effectiveness of PD [25,27]. In addition, elevated triglycerides were associated with reduced RKF, while lower levels of triglycerides correspond with better-preserved RKF [44]. Other lipid markers, such as total cholesterol and LDL, were also associated with RKF and the glucose metabolism, further influencing PD treatment outcomes [44,69]. Both high and low levels of LDL, high AIP, as well as a decreased albumin-to-non-HDL ratio were linked to increased risks of all-cause mortality [22,173,174]. HDL levels were directly associated with peritoneal Kt/V and weekly CrCl [7], while HDL low levels have been consistently associated with early PD withdrawal and a higher risk of technique failure [7,171]. Atherogenic dyslipidemia was significantly associated with intraperitoneal inflammation [86], UFF [24], a higher risk of PD technique failure [7], and long-term mortality [5].

Overall, clinical observations show a significant association between altered lipid profile markers and PD-related outcomes, highlighting the need for managing lipid levels to enhance the efficacy of PD, preserve RKF, and improve patient survival. However, the predominance of retrospective study designs underlines the necessity for prospective research to confirm these findings and explore causal relationships.

## 8. Targeting Dyslipidemia in PD: Available Strategies for Enhancing Peritoneal Membrane Function

As discussed above, the interplay between dyslipidemia, chronic inflammation, oxidative stress, and insulin resistance in patients undergoing PD creates a vicious cycle that accelerates peritoneal membrane deterioration, leading to impaired dialysis efficacy and adverse patient outcomes. Consequently, targeted therapeutic strategies aimed at controlling dyslipidemia may offer significant benefits not only for cardiovascular health, but also for preserving peritoneal membrane integrity and function. However, the dilemma for clinicians managing PD patients with dyslipidemia is the absence of relevant evidence to guide treatment decisions. While lifestyle and dietary modifications and lipid-lowering therapies, such as statins and PCSK9 inhibitors, are recommended for cardiovascular risk reduction in the general population [41,155], the benefits of these treatments in PD patients remain unclear. Despite the apparent need for lipid management in this population, current guidelines do not recommend initiating lipid-lowering therapy in patients already on dialysis, including PD. According to KDIGO guidelines, lipid-lowering therapies such as statins or statin/ezetimibe combinations should only be continued if the patient was already receiving them at the time of dialysis initiation [28]. This recommendation reflects the lack of robust evidence demonstrating a clear benefit of starting dyslipidemia treatment for improving CVD outcomes after dialysis initiation [28,47]. Furthermore, despite some sporadic research [31,32,33], data suggesting the potential benefits of treating dyslipidemia specifically to improve peritoneal membrane function have not been evaluated in randomized clinical trials. The lack of targeted research creates significant uncertainty about whether and how to treat dyslipidemia in PD patients. As a result, clinicians face challenges in decision making, with no clear evidence-based guidelines to guide the prioritization of lipid-lowering interventions for this unique patient population. Below is a summary of the available treatment options, along with their pros and cons.

### 8.1. Lifestyle and Dietary Modifications

In the general population, the initial management of dyslipidemia should focus on non-pharmacological interventions, including dietary modifications and physical activity [175,176]. Among non-dialysis CKD patients, adherence to the Mediterranean diet has been associated with improved lipid profiles [177]. However, there is a lack of randomized clinical trials specifically assessing the impact of dietary interventions on lipid profiles in PD patients [175,178]. Increasing the dietary fiber intake has demonstrated positive effects on mortality rates in PD patients, along with potential benefits such as mitigating constipation, reducing volume and sodium overload, managing hypertension, and alleviating metabolic acidosis [178]. Fiber intake also appears to have favorable effects on the gut microbiome, which could indirectly influence the lipid metabolism. However, the impact of plant-based diets on the total energy and protein intake, as well as their effects on malnutrition, serum electrolytes, and albumin levels, remains unclear and inconsistent in the current data [178].

Aerobic exercises have been shown to improve total cholesterol, HDL, LDL, and triglyceride in patients with hyperlipidemia [179]. Physical activity, tailored to an individual’s capabilities, is also recommended for PD patients [180]. However, the impact of physical activity on lipid profile markers has not been conclusively demonstrated in the PD population [181]. The lack of well-designed research hampers our ability to draw definitive conclusions regarding the impact of increased physical activity on lipid profiles in PD patients. More rigorous studies are needed to validate the potential role of lifestyle and dietary modifications in managing dyslipidemia and improving peritoneal membrane function in this population.

### 8.2. PD Therapy Optimization

Reducing glucose exposure is a critical and the only proven strategy for preserving peritoneal membrane function in PD patients. Alternative PD solutions such as icodextrin, which uses a glucose polymer to provide osmotic pressure without the harmful effects of glucose, have been shown to improve peritoneal ultrafiltration and reduce the risk of peritoneal fibrosis [15,182]. Similarly, amino acid-based solutions can offer nutritional benefits while minimizing glucose absorption, although they are less commonly used due to concerns about their effects on the acid–base balance and protein metabolism [182]. Another glucose-sparing approach involves the use of neutral pH, low-GDP PD solutions. Neutral pH solutions have been shown to decrease oxidative stress markers, lower rates of mesothelial cell apoptosis, and enhance long-term peritoneal membrane function compared to conventional solutions [182,183].

Reducing glucose exposure also aligns with broader goals of metabolic management in PD patients, as it can help mitigate associated dyslipidemia and insulin resistance [71]. Research has shown that modifying PD regimens to reduce glucose exposure can have beneficial effects on patients’ lipid profiles [71]. When one glucose-based PD exchange was substituted with icodextrin, studies observed a reduction in total cholesterol, LDL, and triglyceride concentrations [71]. Incorporating both amino acid-based solutions and icodextrin as part of a glucose-sparing strategy led to improved serum triglyceride, VLDL, and apolipoprotein B levels [74].

However, despite the clear benefits of reducing glucose exposure, the clinical effects of alternative PD solutions on peritoneal membrane function, the preservation of RKF, PD-related infections, and the overall technique and patient survival remain less well-defined [184,185]. Additionally, while glucose-sparing regimens can contribute to metabolic control and a reduced cardiovascular risk, the broader clinical implications, including effects on PD technique survival, require further investigation through well-designed, randomized clinical trials.

### 8.3. Hydroxymethylglutaryl-CoA Reductase Inhibitors

As previously discussed, hydroxymethylglutaryl-CoA reductase inhibitors, or statins, are not routinely recommended for preventing cardiovascular events in the dialysis population due to their minimal or no impact on major cardiovascular outcomes [28]. However, emerging evidence highlights that statins offer multiple benefits in patients undergoing PD, including lowering LDL, reducing systemic inflammation, mitigating peritoneal membrane damage, and reducing all-cause mortality risk [30,31,33].

Experimental studies have demonstrated that statins can alleviate peritoneal membrane damage by targeting inflammation and fibrosis. Specifically, statins have been shown to exert anti-fibrotic effects in peritoneal mesothelial cells by reducing the expression of serum/glucocorticoid-regulated kinase 1 and fibronectin, thus limiting the transition of mesothelial cells into myofibroblasts [186]. Furthermore, statin therapy has been observed to counteract the MMT of HPMCs and reduce ECM accumulation in a PD rat model [31]. In addition, statins stimulate the production of the tissue plasminogen activator and reduce the levels of PAI-1 in HPMCs. This modulation improves the fibrinolytic balance, which helps to prevent fibrosis and preserve the functional integrity of the peritoneal membrane [187]. Atorvastatin, in particular, has been shown to reduce intracellular levels of ROS in rat peritoneal mesothelial cells, thereby protecting against oxidative stress [188]. Furthermore, atorvastatin use diminished structural changes in the peritoneum and the decreased intraperitoneal expression of TGF-β and VEGF [32,189]. Combining rosuvastatin with the angiotensin receptor blocker (ARB) valsartan has been demonstrated to enhance vascular dysfunction more effectively than ARB monotherapy alone [190].

Clinical evidence supports these findings as well. A prospective study found that atorvastatin treatment was associated with the normalization of the phosphate–calcium metabolism, decreased PDE concentrations of inflammatory cytokines (IL-6, IL-10, TNF-α, and MCP-1), the reduced incidence of PD-associated peritonitis, and improved dialysis adequacy [33]. This study concluded that the pleiotropic effects of atorvastatin could contribute to the observed lower all-cause mortality in PD patients.

Additionally, a nationwide retrospective observational propensity-matched cohort study by Kim et al., involving 1596 patients in the statin group and 1596 in the matched control group, demonstrated that initiating statins in statin-naïve dialysis patients was associated with a lower risk of all-cause mortality, though it did not significantly affect the incidence of major adverse cardiovascular events [30]. Complementing these findings, a recent meta-analysis examining the effects of statins in the PD population specifically showed significant reductions in LDL, total cholesterol, and CRP levels [191]. However, this meta-analysis did not find clear evidence of a positive impact on all-cause mortality. The authors suggested that the observed differences might be related to the unique characteristics of dyslipidemia, the inflammatory status, and albumin levels in PD patients compared to those on HD [191].

Altogether, the available evidence underscores the multifaceted benefits of statins in the PD population, extending beyond their traditional lipid-lowering effects. Statins appear to improve peritoneal membrane function, reduce systemic inflammation, and enhance overall patient outcomes. Despite these benefits, further research is needed to fully understand the mechanisms underlying these effects and to establish comprehensive guidelines for the use of statins in the management of PD patients.

### 8.4. Other Pharmacological Treatments

#### 8.4.1. Omega-3 Polyunsaturated Fatty Acids

Omega-3 Polyunsaturated Fatty Acids (PUFAs) are recognized for their beneficial effects on the lipid metabolism and inflammation. For patients undergoing PD, it is recommended to consume 1.3–4 g per day of omega-3 PUFAs to improve lipid profiles [177]. Although studies in the dialysis population are generally limited, omega-3 PUFAs have been shown to notably reduce serum triglycerides, LDL, and inflammation index levels [192]. In patients undergoing PD, omega-3 PUFAs specifically improve triglyceride and HDL levels [193]. However, their role extends beyond lipid modulation and includes significant impacts on peritoneal fibrosis and dialysis efficiency.

In a rat PD model, Tang et al. have shown that the intragastric administration of omega-3 PUFA effectively inhibited peritoneal inflammation and fibrosis by regulating M2 activation and the TGF-β1 signaling pathway [194]. A recent study by Zhang et al. has identified the decreased expression of free fatty acid receptor 4 (FFAR4) in HPMCs and a mouse PD model [195]. Treatment with omega-3 PUFAs restored FFAR4 expression, improving peritoneal fibrosis by inhibiting hyperglycolysis and the MMT via the activation of the FFAR4/CaMKKβ/AMPK pathway and the suppression of mTOR phosphorylation [195].

The cumulative evidence from these studies underscores the potential advantages of omega-3 PUFAs in the management of PD patients, highlighting their role as a valuable adjunctive therapy. However, further research, including larger clinical trials, is needed to confirm these benefits and establish optimal dosing regimens for this patient population.

#### 8.4.2. Sodium–Glucose Cotransporter 2 Inhibitors

Sodium–glucose cotransporter 2 (SGLT2) inhibitors have recently gained attention for their potential role in managing dyslipidemia and peritoneal fibrosis in patients undergoing PD. They have demonstrated significant effects on lipid profiles, including decreases in total cholesterol, LDL, and triglyceride levels, while simultaneously increasing HDL [196,197]. The mechanisms behind these lipid changes are not fully elucidated, but may involve increased lipoprotein lipase activity, shifts in substrate utilization from carbohydrates to lipids and ketone bodies, and alterations in lipid synthesis and transportation pathways [197].

It is crucial to note that the majority of studies on SGLT2 inhibitors have been conducted in non-dialysis CKD populations, and their safety and efficacy in PD patients are not yet fully established. As a result, the effectiveness of SGLT2 inhibitors in specifically managing dyslipidemia in PD patients remains unclear. To bridge this gap, several registered clinical trials are currently investigating the use of SGLT2 inhibitors in PD patients, with a focus on the peritoneal glucose uptake, the mechanisms and safety of SGLT2 inhibition, and its effects on ultrafiltration. These trials are expected to guide the integration of SGLT2 inhibitors into PD treatment protocols.

However, experimental studies have already suggested that SGLT2 inhibitors could offer substantial benefits for PD patients by modulating the glucose uptake from PD solutions and improving peritoneal solute and water transport. HPMCs express glucose transporters such as GLUT1, GLUT3, SGLT1, and SGLT2, which are targeted by SGLT2 inhibitors to reduce glucose absorption and enhance ultrafiltration [198,199]. Experimental models have also shown that SGLT2 inhibitors, such as empagliflozin and canagliflozin, can reduce peritoneal fibrosis, inflammation, and the microvessel density while enhancing peritoneal ultrafiltration [200,201]. These effects are achieved through the inhibition of key pathways, including TGF-β/Smad3 and hypoxia-inducible factor 1 α, along with a reduction in oxidative stress via the Nrf2/HO-1 signaling pathway [200,201].

Clinical studies, although very limited, have shown promising outcomes with the off-label use of dapagliflozin in PD patients, including reduced insulin requirements, improved blood glucose control, and enhanced ultrafiltration volumes, without adverse effects [202]. Despite these encouraging findings, robust clinical trials are necessary to fully confirm the safety and efficacy of SGLT2 inhibitors in PD patients, and their inclusion in PD protocols should be approached cautiously.

#### 8.4.3. Sevelamer

Sevelamer, a non-calcium phosphate binder, is widely used in patients with ESKD to manage hyperphosphatemia [203]. Beyond its phosphate-binding properties, sevelamer has shown potential benefits in the treatment of dyslipidemia in patients undergoing PD [203]. It has been found to reduce serum total cholesterol and LDL and improve overall lipid profiles by binding bile acids in the intestine, which interrupts the enterohepatic circulation of bile acids and subsequently increases the expression of hepatic LDL receptors, leading to the enhanced clearance of LDL cholesterol from the bloodstream [204]. This effect on the lipid metabolism positions sevelamer as a valuable adjunct in the management of dyslipidemia in PD patients.

In addition to its lipid-lowering effects, sevelamer may also exert protective effects on the peritoneal membrane. By binding to endotoxins and bile acids, sevelamer reduces their pro-inflammatory and fibrogenic effects, which may help preserve the peritoneal membrane function over time [205]. Studies have demonstrated that sevelamer can decrease the expression of inflammatory cytokines such as IL-6, IL-8, MCP-1, and TNF-α, which are linked to peritoneal fibrosis [204,206]. Moreover, sevelamer has been shown to reduce AGEs-induced endothelial dysfunction biomarkers in uremic conditions [206].

However, while the initial findings are promising, more extensive clinical trials are needed to confirm the efficacy of sevelamer in managing dyslipidemia and preventing peritoneal fibrosis specifically in the PD population. Such studies should aim to establish optimal dosing, long-term safety, and its potential synergistic effects when combined with other treatments. Until then, the use of sevelamer in PD patients should be tailored to individual patient profiles, taking into account the broader implications of its effects beyond phosphate control.

#### 8.4.4. Proprotein Convertase Subtilisin/Kexin Type 9 Inhibitors

Proprotein convertase subtilisin/kexin type 9 (PCSK9) inhibitors have emerged as a potent therapeutic option for managing dyslipidemia, particularly in patients who do not achieve target lipid levels with statins alone [207]. These monoclonal antibodies target PCSK9, a protein that binds to LDL receptors in the liver, promoting their degradation and reducing the liver’s ability to clear LDL cholesterol from the blood [207,208]. PCSK9 inhibitors significantly increase the number of LDL receptors available to clear LDL cholesterol, resulting in a profound reduction in LDL levels. This mechanism is particularly relevant in PD patients, who demonstrated a direct association between plasma PCSK9 levels and LDL concentrations [168]. The ALIDIAL study, which administered alirocumab f at a full dose of 150 mg every 2 weeks for 12 weeks to patients on KRT, showed a significant reduction in LDL levels [209]. Additionally, the levels of ceramides, sphingomyelins, and cholesterol esters were also significantly reduced after the treatment.

Although recent studies suggest the potential benefits of PCSK9 inhibitors in improving endothelial function and reducing inflammation [208,210], the ALIDIAL study has not identified an influence on inflammatory markers in their dialysis cohort [209].

To better define the role of PCSK9 inhibitors in PD, clinical trials focusing on their impact on lipid profiles, endothelial function, chronic inflammation, and overall patient outcomes in PD are necessary.

#### 8.4.5. ApoA-I Mimetic Peptides

ApoA-I mimetic peptides are designed to replicate the beneficial properties of ApoA-I and have been extensively studied for their role in CVD prevention [211]. Their primary functions include promoting cholesterol efflux from cells, remodeling HDL particles, sequestering oxidized lipids, and activating anti-inflammatory processes in macrophages [212]. One of the most researched ApoA-I mimetics, the 4F peptide, has demonstrated the ability to reduce early atherosclerosis in animal models, enhance the formation of pre-β HDL, improve HDL function, and lower inflammation and oxidative stress [211,212].

While direct clinical evidence for the use of ApoA-I mimetic peptides in PD is still emerging, animal models of PD have shown that these peptides can reduce peritoneal inflammation and oxidative stress, decrease the MMT of peritoneal mesothelial cells, and slow the progression of peritoneal fibrosis [122]. These findings suggest that ApoA-I mimetics could play a dual role in managing dyslipidemia and preserving peritoneal membrane function.

However, challenges remain in translating the benefits seen in experimental models to clinical use. Optimizing peptide delivery and demonstrating efficacy in human trials are crucial steps that need to be addressed. While the potential of ApoA-I mimetic peptides in managing dyslipidemia and reducing peritoneal fibrosis is promising, more research is needed to determine their potential role in managing complications of PD.

### 8.5. Gut Microbiota Modulation by Pre-, Pro-, and Synbiotics Supplementations

Modulating the gut microbiota through prebiotics, probiotics, and synbiotics represents a promising strategy to address dyslipidemia in patients with ESKD [110,213]. Prebiotics are non-digestible fibers that promote the growth of beneficial gut bacteria, probiotics are live microorganisms that confer health benefits when administered in adequate amounts, and synbiotics combine both prebiotics and probiotics to enhance gut health synergistically [110,213]. Biotics administration has been shown to increase HDL levels in the dialysis population [214]. However, the potential benefits of gut microbiota modulation extend beyond lipid management and reducing CRP, and indoxyl sulfate, IL-6, TNF-α, and oxidative stress markers in patients with ESKD on dialysis have been found in two recent meta-analyses [214,215].

Nonetheless, despite growing interest, the specific evidence base for PD patients specifically remains limited, with only two randomized studies directly investigating the effects of these interventions in this population. Pan et al. conducted a randomized clinical trial with 98 PD patients to assess the impact of probiotic supplementation on the gut microbiota and overall health outcomes [216]. Patients received a probiotic containing *Bifidobacterium longum*, *Lactobacillus bulgaricus*, and *Streptococcus thermophilus* for 2 months. The study found significant reductions in serum CRP and IL-6 levels, indicating decreased systemic inflammation, along with improvements in serum albumin, the upper-arm circumference, and the triceps skinfold thickness, partially improving malnutrition and quality of life. There was also a positive shift in the gut microbiota, with increased beneficial bacteria and reduced uremic toxins [216].

Wang et al. investigated the potential of probiotics to reduce PD-related peritonitis in a randomized trial with 39 PD patients [217]. A specific probiotic formulation was administered for 3 months, resulting in a lower rate of peritonitis episodes compared to the control group. The study also found significant reductions in serum endotoxin, TNF-α, and IL-6 levels, along with an increase in IL-10. The preservation of RKF was also observed, highlighting the potential benefits of probiotics in infection prevention and the maintenance of RKF in PD patients [217].

Despite the encouraging findings from initial studies, further research is needed to validate these results in larger, well-designed clinical trials. Additionally, the long-term safety and efficacy of pre-, pro-, and synbiotic supplementation in PD patients must be established. Exploring personalized approaches that consider individual microbiota profiles and tailoring interventions accordingly could enhance the therapeutic potential of these strategies.

## 9. Conclusions and Future Directions

Together, dyslipidemia is a prevalent and multifaceted disorder in patients undergoing PD, traditionally considered primarily for its impact on cardiovascular outcomes. However, it may also play a critical role in the structural and functional integrity of the peritoneal membrane, influencing PD-related outcomes such as the peritoneal ultrafiltration rate, RKF, PD technique survival, and overall patient survival. Potential mechanisms through which dyslipidemia may harm the peritoneal membrane include intracellular lipid accumulation, oxidative stress, inflammation, endothelial dysfunction, and interactions with the RAAS. In addition, gut microbiota dysbiosis has emerged as a possible contributor to both dyslipidemia and peritoneal membrane damage. Current strategies of dyslipidemia management do not fully address the unique metabolic challenges of PD patients. Addressing dyslipidemia with a broader perspective could provide significant benefits in improving the quality of care and patient survival in this unique patient population.

Future research should focus on defining standardized criteria for dyslipidemia in PD patients, elucidating the specific lipid abnormalities that are most significantly associated with peritoneal membrane integrity, and developing targeted interventions. The discussed therapeutic approaches show promise, but require validation through well-designed clinical trials. The trials should also evaluate the potential of personalized therapies that account for factors such as RKF, comorbidities, and genetic predispositions. An understanding of the relationship between dyslipidemia and peritoneal membrane dysfunction will pave the way for improved therapeutic strategies, ultimately enhancing the quality of life and survival of PD patients.

## Figures and Tables

**Table 1:** Association Between Lipid Profile Markers and PD-related Patients’ Outcomes.

Study	Study Design and Patients	Associated Lipid Profile Markers	Key Findings
Lin et al., 2018 [44]	Retrospective, nationwide population-based study of 8022 patients	TC	Patients with preserved RKF (renal CrCl > 2 mL/min/1.73 m^2^) had significantly lower triglyceride levels compared with anuric patients. A U-shaped mortality curve was observed when TC was lower than 150 mg/dL and higher than 250 mg/dL in PD patients without RKF.
Luo et al., 2018 [171]	Retrospective, single-center study, 1444 patients	HDL	Low HDL predicted very early PD withdrawal (during the first 90 days).
Stepanova et al., 2019 [7]	Prospective single-center study, 40 patients	Atherogenic dyslipidemia	HDL levels were directly associated with peritoneal Kt/V and weekly CrCl; TG had an inverse correlation. Atherogenic dyslipidemia was significantly associated with a higher risk of three-year technique failure.
Stepanova et al., 2020 [86]	Cross-sectional, single-center study, 40 patients	Atherogenic dyslipidemia	An atherogenic lipid profile was significantly associated with a high concentration of MCP-1 in PDE.
Wan et al., 2021 [91]	Retrospective study of 291 patients	TG	Serum TG ≥ 1.4 mmol/L at the initiation of PD was associated with early-onset peritonitis, technical failure, and overall mortality risk.
Liu et al., 2022 [172]	Retrospective cohort study, 210 patients	HDL	Time-averaged HDL was an independent protective factor for technique failure and all-cause mortality.
Wu et al., 2022 [173]	Retrospective, multi-center real-world cohort study, 3565 patients	LDL	Both higher levels of LDL (>2.60 mmol/L) and lower levels of LDL (<2.26 mmol/L) were associated with increased risks of all-cause mortality.
Feng et al., 2022 [5]	Retrospective cohort study, 2939 patients	Atherogenic dyslipidemia	Hyperlipidemia at the beginning of CAPD was associated with a high risk of long-term mortality.
Yang et al., 2022 [27]	Retrospective cohort study, 276 patients	TG, HDL	Higher TG and lower HDL levels were correlated with technique failure risk, and TG level is an independent predictor of technique failure in the adjusted model.
Huang et al., 2022 [25]	Retrospective cohort study, 66 patients	TG	TG level ≥ 1.7 mmol/L was significantly associated with treatment failure of PD-related peritonitis.
Honda et al., 2022 [23]	Retrospective cohort study, 113 patients	HDL	HDL at PD initiation was independently associated with a change in renal Kt/V during the first year after PD initiation.
Xu et al., 2022 [69]	Retrospective single-center study, 2384 patients	TC, LDL, TG	TC, TG, and LDL levels were significantly associated with baseline fasting plasma glucose > 7 mmol/L, total Kt/V, RKF, and glucose load.
Zhang et al., 2022 [89]	Retrospective cohort study, 1013 patients	HDL	Patients with both diabetes and low HDL levels were at higher risk for PD-related peritonitis.
Deng et al., 2023 [22]	Retrospective cohort study, 2682 patients	AIP	Gradually increased AIP was independently associated with all-cause mortality.
Lu et al., 2023 [122]	Retrospective cohort study, 81 patients	ApoA/HDL ratio	A decreased ApoA/HDL ratio is significantly associated with a rapid decline in peritoneal function.
Stepanova et al., 2023 [24]	Cross-sectional, bi-center cohort study, 114 patients	Atherogenic dyslipidemia	Dyslipidemia was significantly associated with increased intensity of oxidative stress and UFF.
Xie et al., 2024 [174]	Retrospective cohort study, 1954 patients	Non-HDL	A decreased albumin to non-HDL ratio is an independent risk factor for all-cause mortality.

Abbreviations: AIP, atherogenic index of plasma; ApoA, apolipoprotein A; CAPD, continuous ambulatory peritoneal dialysis; CrCl, weekly peritoneal creatinine clearance; HDL, high-density lipoprotein cholesterol; Kt/V, a measure of dialysis adequacy; LDL, low-density lipoprotein cholesterol; MCP-1, monocyte chemoattractant protein-1; PD, peritoneal dialysis; PDE, peritoneal dialysis effluent; RKF: residual kidneyfunction; TC, total cholesterol; TG, triglycerides; UFF, ultrafiltration failure.

## Data Availability

Not applicable.
